# Homogeneous image-based digital immunoassays with high error tolerance

**DOI:** 10.1038/s44303-026-00164-9

**Published:** 2026-05-04

**Authors:** Darren B. McAffee, Qiang Hu, Assame Arnob, Hung-Jen Wu, Jay T. Groves

**Affiliations:** 1 ilytica, LLC., San Francisco, CA USA; 2 Department of Chemical Engineering, Texas A&M University, College Station, TX USA; 3 Department of Chemistry, University of California, Berkeley, CA USA

**Keywords:** Biological techniques, Biomarkers, Biotechnology, Nanoscience and technology

## Abstract

There is a significant global health need to translate more in vitro diagnostic tests from clinical laboratories to field-based applications, including point-of-care and self-administered test formats. These applications typically require smaller sample sizes, limit sample processing and measurement capabilities, and introduce greater handling variability. Error tolerance is one of the most critical factors for successful field-based assay design. Here, we examine machine-learning (ML) strategies to enhance the error tolerance of image-based nanoparticle immunoassays. Random dispersions of nanoparticles were imaged in microliter sample volumes, and images were processed to determine analyte concentrations based on nanoparticle appearance. Assay performance was characterized using two common blood analytes: C-reactive protein and anti-SARS-CoV-2 IgG. We compare the results from conventional image analysis, a hybrid ML-conventional approach based on pixel segmentation, and end-to-end image regression using a targeted regularization strategy. Using serum samples from SARS-CoV-2 positive individuals, the segmentation-based approach enabled binary classification with 96% specificity and 90% sensitivity, matching seroconversion rates. The end-to-end regression model achieved superior quantitative performance (5.2 ng/mL), approaching ELISA-level detection range (0.01–10 ng/mL, depending on capture antibody affinity) in a single 30 min workflow without sample preprocessing. The limit of detection for digital molecular assays is not fixed, and we perform a theoretical analysis showing how adjusting particle counts and polydispersity can achieve arbitrary sensitivity down to the Poisson limit. Training images for the full image regression approach required only a single label—the analyte concentration—eliminating labor-intensive pixel-level labeling. Ultimately, the image-based readouts significantly improved dynamic range, sensitivity, and reproducibility over conventional readouts.

## Introduction

Enzyme-linked immunosorbent assays (ELISAs) are the clinical gold standard for sensitive and quantitative biomarker detection, but they require centralized laboratory infrastructure, multiple wash steps, and turnaround times that limit their use outside clinical laboratories. In contrast, lateral flow assays (LFAs) enable rapid, low-cost testing at the point of care, but typically sacrifice analytical sensitivity, quantitative accuracy, and reproducibility, particularly in complex matrices such as whole blood. This gap between laboratory-grade performance and field-deployable simplicity remains a central challenge in the development of accessible diagnostic technologies.

The digitization of immunoassays—shifting from bulk signal measurements to individual particle-by-particle analyses—is a rapidly advancing trend in biomarker detection^1^. This analog-to-digital transition in molecular detection mirrors, in many ways, the analog-to-digital transition in the electronics industry. Similarly, it holds great promise to achieve higher sensitivity, precision, and especially error resistance in molecular diagnostics. Techniques like Single Molecule Array (Simoa)^2^ or Single Molecule Counting (SMC)^3^ assays achieve ultrahigh sensitivity by isolating individual target molecules within femtoliter-sized wells or droplets, respectively, thereby digitizing the assay. Existing digital assay methods still require precision-engineered well arrays, droplet generators^4^, or high-end imaging setups^5,6^ to minimize background noise from non-specific binding, optical scatter, and image artifacts—all of which can severely impact accuracy. Error tolerance isn’t an automatic benefit of molecular assay digitization. However, high pixel count images of particle-based assays include vast amounts of information beyond the analyte detection signals themselves. This additional information can be used to identify and filter noise and error sources that would otherwise be inexorably blended in the assay readout^7^.

While sensitivity improvements have historically driven innovation in immunoassay development, reliability and error tolerance are often more critical for POC and self-testing^8–13^. Many clinically relevant protein biomarkers are present at picomolar (pM) or higher concentrations^14^, which are readily detectable by many immunoassay formats. Diagnostic errors arising from pre-analytical factors, such as sample contamination and improper handling, and post-analytical factors, such as ambiguous readout interpretation, present greater challenges to field-based assay development^15–18^. In principle, an image-based format could allow an ML model to detect certain pre-analytical errors like hemolysis or low sample volume that would otherwise be convolved in the sample readout. The real-world coefficient of variation (CV) of an assay is generally of greater importance than the limit of detection (LOD) established in controlled settings^8,19,20^. There is a substantial need for technology advancements that increase assay error-tolerance and simplicity. In this study, we explore ML-driven error filtering to increase error tolerance and assay performance in image-based digital immunoassays.

We examine biomarker assays based on the clustering behavior of gold nanoparticles (AuNPs)^21^, but the strategies employed are broadly applicable to other signal reporters such as shaped plasmonic nanoparticles^22,23^, colloidal particles^24–27^, fluorophores^6^, or many other reporter types^28^. Functionalized AuNP assays to detect CRP and anti-SARS-CoV-2 IgG were performed on microliter sample volumes of serum and whole blood. Direct imaging of nanoparticle clustering by darkfield microscopy provides measurable improvements over bulk spectroscopic readouts, increasing both sensitivity and dynamic range. While standard image processing algorithms^29^ functioned adequately in limited circumstances, they were often brittle and required meticulous sample preparation to minimize error.

**Fig. 1:**
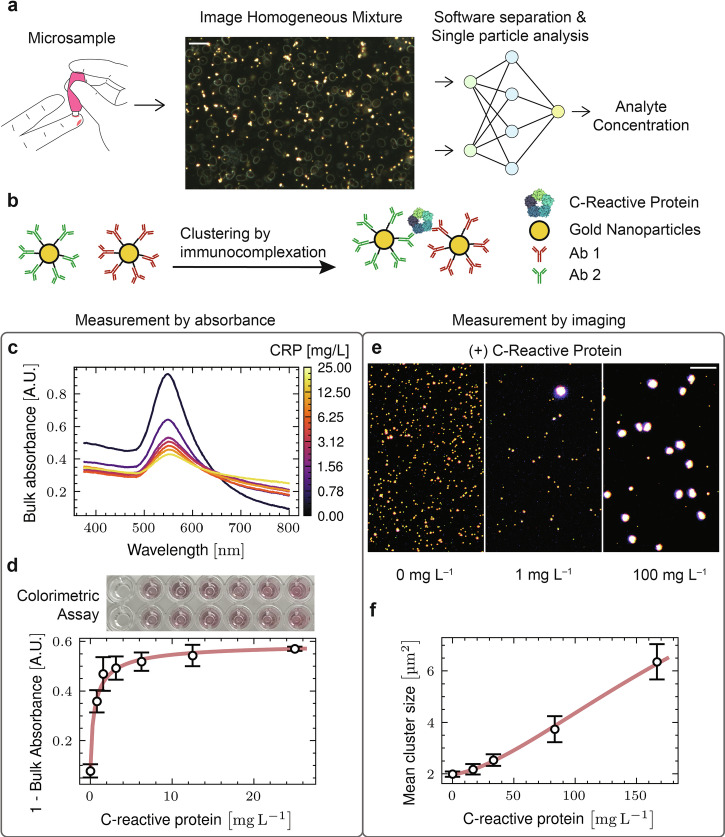
Imaging improves dynamic range over spectroscopic approach. **a** Schematic of the simplified workflow of a digital assay using random particle arrays of gold nanoparticles imaged in-situ with sample. Scale bar 20 µm. Neural network graphic adapted from Wikimedia Commons artwork^45^ **b** Design of particle aggregation assay towards C-reactive protein. **c**/**d** Traditional absorbance-based methods for inference of analyte concentration. **c** Spectra of samples with increasing CRP concentrations with associated decrease in 555 nm absorbance and simultaneous red-shifting. **d** Titration of CRP concentration against spectral shifts. **e** Example images using 20x darkfield to image the nanoparticle aggregates in the presence of increasing CRP concentration. Scale bar is 10 µm. **f** Titration of CRP concentration against quantification of aggregate size. Analysis was on raw images, but images shown here are contrast enhanced. Error bars represent SEM.

We sought to improve image-based digital assay performance using two different ML strategies. First, an ML-assisted strategy based on pixel segmentation to filter out image artifacts prior to conventional image analysis offered improvements, but still required tedious training. Finally, by coupling a modified ResNet image classification model^30^ with a targeted regularization strategy, we efficiently trained models using only a single label—analyte concentration—eliminating the need for pixel-level labeling. Effective training could be accomplished with ~10,000 images and a single GPU. This end-to-end image regression approach differs substantially from conventional or hybrid ML analyses in that it requires no user-defined criterion to interpret images. All aspects of the image containing useful information are utilized, providing substantial performances improvements in high error settings. With full ML analysis, clinical laboratory level performance (e.g. comparable to ELISA) was achieved with an extremely simple mix-and-measure workflow on whole blood microsamples (Fig. 1a). These findings highlight the potential of image-based digital immunoassays to leverage rapidly developing ML capabilities. The resulting reduction in hardware and workflow complexity while maintaining high diagnostic accuracy is a crucial step toward developing next-generation POC diagnostics that are robust, widely accessible, and ready to meet global health needs.

## Results

### Comparison of spectrographic and image-based readouts

Biomolecular assays based on functionalized AuNPs are widely used in diagnostics, including lateral flow assays (LFAs)^31^ and bulk solution colorimetric assays^21^. In these formats, capture agents linked to the AuNP (e.g. antibodies, aptamers, nanobodies, etc.) bind the target analyte. Two capture agents binding distinct epitopes on the target will lead to crosslinking or localization of the particles in an analyte-dependent manner (e.g. sandwich immunoassay). Alternatively, multivalent targets can crosslink particles with even a single capture agent^32^. In LFAs, signal is produced as enhanced optical density by concentrating nanoparticles at a designated test strip location whereas signal in solution-based assays is detected as color or light scattering changes caused by nanoparticle oligomerization. LFAs and solution-based assays are generally analyzed by bulk optical readout, in which individual particles are not resolved—only an average optical response from all particles is read. However, individual nanoparticles can be readily imaged by simple techniques such as dark-field microscopy^33–35^. Image-based assays employ microscopy to directly resolve the entire dispersion of nanoparticles, offering the possibility to individually evaluate each nanoparticle or cluster. Image analysis steps are then employed to interpret nanoparticle features from the images (e.g. mean nanoparticle cluster size, color, or brightness), which can be related to analyte concentration^36,37^. An important advantage of image-based assay readout is the possibility to filter out various forms of error (e.g. dust, bubbles, pre-aggregated particles etc.) from the final analyte concentration determination based on definable image characteristics^7^.

To assess the performance advantages of image-based readout, we first examine a classic two-antibody sandwich AuNP immunoassay for CRP. AuNPs (80 nm diameter) were conjugated with two CRP-specific antibodies (Fig. 1b). Clustering of anti-C-reactive protein particles by the CRP analyte results in a red-shifted absorption-scattering peak, which can be seen by eye and read quantitatively by UV-Visible spectroscopy^21^. Assays were run by incubating anti-CRP particles with CRP in plasma or whole blood at varying concentrations. As CRP concentration increased, the 555 nm peak in the absorption-scattering spectrum decreased (Fig. 1c). Quantification of the 555 nm absorbance peak magnitude provides a robust readout for the assay but it has a limited dynamic range, due partly to larger aggregates falling out of solution as well as the fact that spectral changes with increasing aggregate size become less pronounced (Fig. 1d). When AuNPs from the same sample solutions are directly imaged (Fig. 1e), numerous aggregates are visible and quantifiable with straightforward image processing techniques^29^. Mean cluster size provides a robust linear response to CRP concentration that extends more than 30 times beyond the saturation limit of the colorimetric assay (Fig. 1f). The imaging readout readily covers the clinical concentration range for CRP diagnostics ( < 1 mg/L to 200 mg/L) using the same particles that failed to cover this range in bulk spectroscopic readout.

**Fig. 2:**
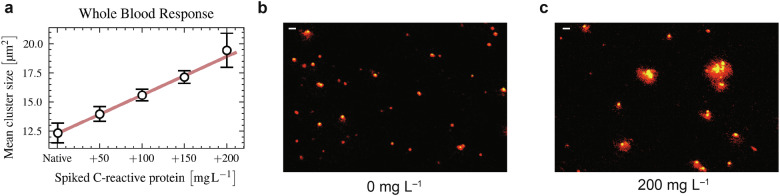
Image-based CRP assay in whole blood. Human blood samples, containing native (endogenous) analyte, were spiked with exogenous C-reactive Protein. **a** Plot of mean cluster size of nanoparticles against added concentration of CRP in blood samples. Error bars represent SEM. **b** Representative image of baseline nanoparticle clustering (no CRP added) in response to endogenous CRP. **c** Representative image of nanoparticle clustering in the presence of endogenous +200 mg/L exogenous CRP. Scale bars are 6 µm. Analysis was on raw images, but images shown here are contrast enhanced.

Additionally, we demonstrate that an image-based approach to analyte inference can be performed by direct imaging of nanoparticle-sample mixtures—without wash or separation steps (Fig. 2). Commercial human blood samples were spiked with exogenous C-Reactive Protein, incubated for 30 min, and imaged. The resulting images contained nanoparticle clusters with noticeable red-halo effects due to hemoglobin (Fig. 2b, c). Despite this, after tuning the image processing parameters (threshold, gaussian blur radius, etc.) nanoparticles could be readily isolated. A clear linear response was observed between the amount of CRP added and the mean cluster size of the functionalized nanoparticles. This demonstrates that the matrix effects of blood, which typically contaminate and ruin most assays, can be mitigated by the image-based approach if the algorithm is properly tuned. The inter-day coefficient of variation (CV) was 5.03%. A notable advantage of the image assay approach is its ability to handle complex samples, such as whole blood, without sample preprocessing.

### ML-assisted analysis based on pixel segmentation

While conventional image analysis based on predefined criterion can be used to perform a rough separation of image artifacts from functional nanoparticles, a tremendous amount of information about the sample remains unused with such methods. Here, we examine an ML-assisted image analysis strategy based on pixel-wise segmentation to computationally separate genuine signal from artifact. Various ML algorithms can learn to do pixel-wise segmentation and there exist open-source software packages enabling facile training^38,39^. In this hybrid format, ML is first used to separate out error sources in images, then a conventional readout (e.g. mean cluster size) is performed on the ML-filtered images to infer analyte concentration.

**Fig. 3:**
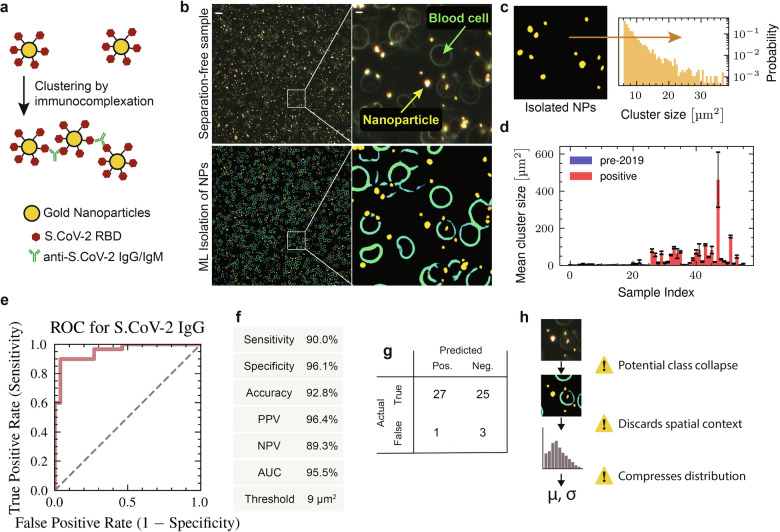
A hybrid segmentation-counting approach for a serological assay. **a** Design of gold nanoparticle assay; aggregation of particles was induced by the presence of antibodies against the RBD of SARS-CoV-2. **b** Example images using 20x darkfield to capture nanoparticle aggregation in whole blood samples. The probability maps for different classes (gold nanoparticle, cell, background) produced by the segmentation model are displayed on bottom while the original image is on top. Scale bar in widefield image is 20 µm and 2.5 µm on inset. **c** Post-processing of cluster size shows an approximate power law distribution. **d** Quantitation of cluster size on biobank sample from two patient groups: one 3 weeks convalescent from COVID-19 (positive) and pre-2019 samples (negative). **e** Plot of the ROC curve for the classification assay in (**d**). **f** Metrics of classification assay when classifying mean nanoparticle aggregates ≥ 9 µm^2^ as positive. **g** Confusion matrix of classification assay. **h** Schematic demonstrating the potentially lossy steps of analysis for the hybrid approach (segmentation + conventional).

We characterized this ML-assisted analysis on AuNP assays for anti-SARS-CoV-2 IgG in patient samples. AuNPs were functionalized with a single capture protein, SARS-CoV-2 RBD, here referred to as RBD-functionalized particles (Fig. 3a). These RBD-functionalized particles exhibited robust aggregation in the presence of immunoglobulins (G and E type) specific to SARS-CoV-2 RBD (Supplementary Fig. 1a, b). Training images were generated using the pixel classification workflow in *ilastik*^38^ and used to train random forest (RF) ML algorithms (see Methods). Although the resulting RF models could be trained quickly with minimal data, they lacked robustness and failed to generalize beyond 8–10 images. To address this limitation, we built an ensemble of seven RF models, each trained on 5–6 annotated images ($\approx \mathrm{50,000}$ particles per image) representing a range of sample types (serum, blood, and PBS) as well as varying degrees of defocus. This ensemble approach helped maintain model coherence and captured greater variability. The combined output — 45 fully pixel-labelled high-resolution (4 K) images — served as the training dataset for a U-net^40^. We applied standard data augmentation techniques from the PyTorch library to enhance segmentation performance (see Methods for detailed descriptions). As a result, the U-net significantly outperformed the directly generated RF models in terms of generalization and was able to rapidly segment images into relevant classes such as background, nanoparticles, dust, and debris. After pixel-wise classification on the blood samples and removal of erroneous objects (Fig. 3b), the mean cluster size of the nanoparticles can be quantified and mapped to analyte concentration (Fig. 3c).

We used this trained pixel segmentation algorithm to quantify anti-SARS-CoV-2 IgG in patient samples. A total of 56 samples were tested, including 26 true negative (pre-2019) and 30 true positive (PCR positive, 3-week convalescent) samples. The patient samples were imaged in an open air microwell format, which introduced high levels of conventional error including bubbles, dust, as well as optical distortions due to the meniscus of the well (Supplementary Fig. 1c). Despite these challenges, the digital molecular assay robustly classified positive COVID-19 samples (Fig. 3d). The segmentation assay provided robust classification statistics (Fig. 3e−g) including 96% specificity and 90% sensitivity—which matches the ≈88% seroconversion rate of COVID-19 positive patients that is typically found^41,42^.

This use of pixel-segmentation for error filtering followed by traditional analysis still relies on predetermined particle features (e.g. cluster size, color, brightness, etc.) in the final image analysis step to infer analyte concentration. A vast amount of analyte-dependent information contained in the images is still left unutilized by such an approach (Fig. 3h). We hypothesized that an end-to-end image regression approach, which maps a full sample image to analyte concentration, could potentially learn which features of the nanoparticles are most important while also learning to ignore optical distortions and other artifacts.

### End-to-end image regression for full ML assay interpretation

**Fig. 4:**
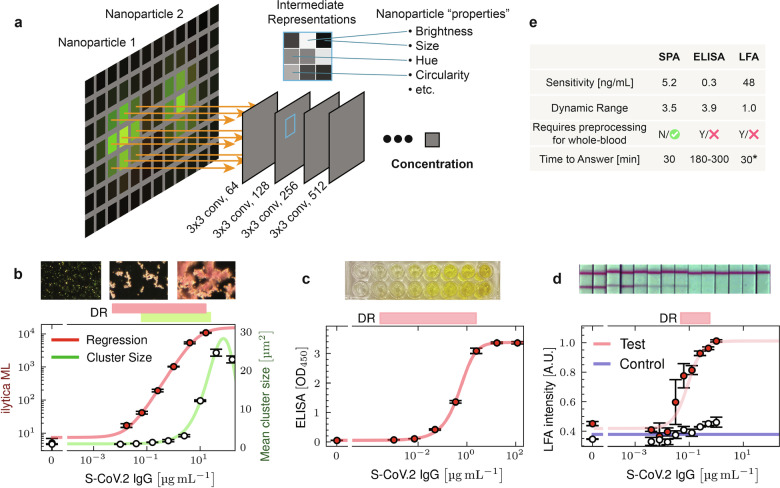
An end-to-end regression model is comparable to ELISA, but with a single incubation step. **a** Schematic of how the end-to-end machine learning model works. The image is processed by several convolutional layers. Deeper layers can capture more abstract properties of the particles and allow surrounding context to influence outcomes. **b** Titration of anti-SARS-CoV-2 IgG against the output of the end-to-end ML model compared to the output of the hybrid segmentation + post-processing model. **c** Titration of anti-SARS-CoV-2 IgG using a commercial ELISA kit. **d** Titration of anti-SARS-CoV-2 IgG using a Universal Lateral Flow Kit with the same particles used in the imaging assay (**b**). **e** Table comparing the single-particle analysis (SPA) method from (**b**), the ELISA method from (**c**) and the LFA method from (**d**).

We developed a full end-to-end image regression approach for assay interpretation by training a modified ResNet-18 model^30^ on 10,000 images labelled only with analyte concentration (Fig. 4a). Approximately 55 non-overlapping images were recorded on each of 182 samples consisting of various concentrations of anti-SARS-CoV-2 IgG spiked in human serum. The labels corresponded to a titrated dataset with 7 concentrations (0, 16, 64, 256, 1024, 4096, and 16384 ng/µL anti-SARS-CoV-2 IgG). The dataset was split into testing and training groups (164 training samples, 18 test samples). Separation by sample (instead of just image) proved important to ensure that model generalization comes from nanoparticle properties rather than sample specific artifacts; several types of sample error span multiple images (e.g. nanoparticle adherence patterns or extended scratches on the substrate).

For our initial attempt, the final layer of the ResNet model consisted of a collection of neurons, corresponding to the number of classes the model is intended to predict (e.g. the 7 trained concentrations). Using this classification approach, we trained the model to predict which labelled concentration the unknown sample image belonged to. While effective, this required additional interpolation methods to infer sample concentrations between the classifications. We next tested a direct regression architecture, where the final layer of the ResNet-18 had a single output neuron intended to only predict the log of the analyte concentration. This single-neuron output strategy, using an L1 regression loss on log(concentration), had significantly better performance. Concentration estimates were made from the mean prediction of 20 images from the sample.

The results from applying the regression model to the testing samples, with known concentrations of anti-SARS-CoV-2 IgG spanning the titration range, are shown in Fig. 4b (red trace). Results from the hybrid ML approach (pixel-segmentation followed by conventional feature processing) are also plotted in Fig. 4b (green trace) for comparison. While the hybrid ML-conventional approach does remove sources of error, it is clearly limited by only analyzing a handful of properties of the nanoparticles, such as brightness and size. While there is no direct map between human and machine interpretation, we speculate that the full regression model relies on a spectrum of image properties that shifts with analyte concentration. For example, properties such as particle density and color ratios are likely more important at lower analyte concentrations, while other properties such as aggregate size and void area are more heavily weighted at higher concentrations. The model is not restricted to following one parameter across the entire detection range. We quantified the assay sensitivity using a fitted Hill function in conjunction with the measurement error around the blank and lowest measured concentration to estimate the limit of detection^43^. The regression model achieved near-ELISA capabilities on a first attempt with a sensitivity of 34.7 pM (5.2 ng/mL) and a dynamic range of 3.5 orders of magnitude—beating the hybrid ML-conventional model by more than an order of magnitude in both characteristics.

For benchmark comparisons, similar anti-SARS-CoV-2 IgG titration results were obtained from traditional ELISA (representing the clinical lab-standard) and LFA (representing the POC-standard) assays. The ELISA was performed from a commercial testing kit (Thermofisher) and achieved a sensitivity of 2 pM (0.3 ng/mL) with a dynamic range of 3.9 orders of magnitude (Fig. 4c). For a direct comparison to LFA, we created an in-house LFA using the same RBD-functionalized particles as were used in the image-based experiments using a universal LFA kit (Abcam). LFA test strips were imaged and the relative intensity of test and control strips were assessed using a laboratory scanner (see Materials and Methods). While the LFA achieved a respectable sensitivity of 0.32 nM (48 ng/mL), it had a poor dynamic range with barely 1 order of magnitude (Fig. 4d), limiting its use as a quantitative assay. Moreover, our use of a laboratory scanner to quantify the LFA results likely improved its performance beyond typical POC applications.

In addition to core assay metrics like sensitivity and dynamic range, there are other important distinctions between the overall formats of the assays. For blood-based testing, both ELISA and LFA usually require plasma/serum separation steps to avoid signal degradation from whole blood. In contrast, an image-based ML approach can be used directly on whole blood, reducing the hardware complexity required. LFA is noted for its rapid turnaround, and image-based digital assay workflows have shown similar turnaround times. Both LFA and image-based digital assays are limited by the primary incubation time between capture and target proteins, which is governed by reagent on and off rates with target. ELISA typically has a significantly more complex workflow, taking 3–5 times longer. These comparisons are summarized in Fig. 4e.

### Sensitivity of cross-linking digital assays

Detection limits in image-based readout of nanoparticle assays are intrinsically at the single molecule level^34^, offering the potential to vastly surpass bulk readout sensitivity. Practically, the overall assay sensitivity limit in an imaging readout is determined by the number of definitive analyte molecular detection events and the ability to distinguish these from nonspecific signals. Analysis of more particles effectively increases the number of trials, thereby reducing noise through counting statistics. In contrast, for bulk measurements such as ELISA, increasing the sample volume does not inherently improve sensitivity. In fact, it can dilute the analyte concentration or introduce additional background noise, potentially lowering the signal-to-noise ratio and making it more difficult to detect low-abundance targets. In the following, we provide a general analysis relating measurable physical properties of the nanoparticle sensors to corresponding assay sensitivity over a broad range of digital assay implementation conditions. The results detailed below illustrate how assay sensitivity can be adjusted, with almost no limit, by adjusting the number of particles utilized in the assay. These analyses apply generally to digital molecular assays. ML error filtering strategies increase both the number of useable particles as well as the fidelity of signal discrimination.

**Fig. 5:**
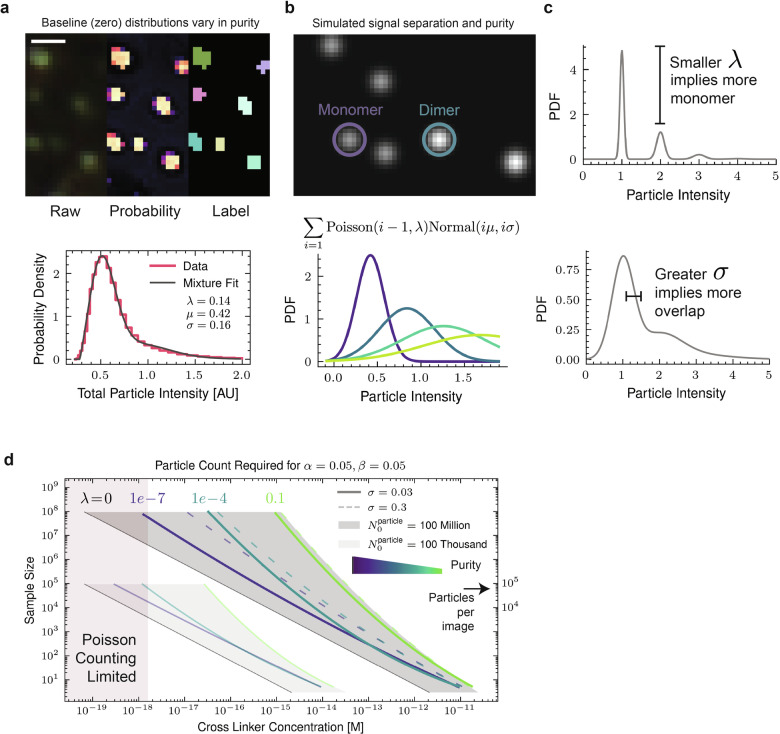
Particle based assays have tunable sensitivity based on counts. **a** Example image of gold nanoparticles with zero analyte (i.e. baseline). Scale bar is 1.3 µm. A histogram of particle intensities is shown below along with a model fit. The model used in the histogram is described by (**b**) A Poisson weighted mixture model of a series of convolved normals is used to approximate a starting particle distribution. Simulated images showing the intensities of monomer and dimer is shown on top. **c** Graphs demonstrating how the different parameters of the mixture model influence total particle-intensity distributions of the aggregates. **d** Line plot of scaling laws (sample size required for a given limit of detection) determined by a Monte Carlo approach.

To assess sensitivity scaling laws for digital assays we consider two critical assay features: particle polydispersity and intrinsic optical contrast. While this analysis focuses on particle clustering assays, it is generally applicable to any droplet or bead digital assay format. We approximate the starting distribution (Fig. 5a) to consist of monomers with a Gaussian intensity distribution, and pre-existing aggregates (dimers, trimers, etc.) are modeled by convolving this distribution with itself. The quality of the starting particle dispersion is thus characterized by the monomer-dimer contrast, governed by $\sigma$, and the fraction of pre-existing aggregates $\left({\rm{\lambda }}\right)$, see Fig. 5b, c. The key question to determine analyte detection limits then becomes: when analytes crosslink particles from some baseline distribution, how many particles must be sampled to reliably detect a difference? Using a Monte Carlo method coupled with likelihood ratio testing we determined how this detection limit depends on ${\rm{\sigma }}$ and ${\rm{\lambda }}$. This was done by sampling from a theoretical baseline particle-intensity distribution and a crosslinked particle-intensity distribution, then computing the likelihood ratio. For the experimental assays described in this study, 5 μL serum samples were mixed with 20 μL particle solutions for a final testing mixture of 25 μL containing $\approx 300$ million AuNps, so the upper limit of particles sampled from these distributions in our simulations was rounded to 100 million. The functionalized AuNPs used in this study had a baseline particle-intensity distribution with ${\rm{\sigma }}/{\rm{\mu }}=0.38$ and ${\rm{\lambda }}=0.14$ (Fig. 5a). Using these particles, the full-ML assay achieved $34.7{pM}$ sensitivity by sampling $\approx 1$ million particles. This realized sensitivity is of similar magnitude as predicted from the baseline particle-intensity distribution alone. Higher precision nanoparticle manufacturing and purification processes can produce particles with significantly lower polydispersity^44^, which can dramatically improve assay sensitivity. However, an even larger sensitivity improvement is achieved by lowering the number of particles in the sample incubation (increasing the fraction of particles that capture analyte). Reducing particle number comes at a cost of incubation time and potentially the need for re-concentration methods.

We calculated scaling curves that map the number of particles that must be sampled to achieve desired analytical detection limits for various particle properties $\left({\rm{\sigma }},{\rm{\lambda }}\right)$ (Fig. 5d). The assay particles used in this study sit just to the right of the light green line. Assay sensitivity can be improved using higher precision manufacturing and purification processes to reduce polydispersity, which can get down to ${\rm{\lambda }}=1\times {10}^{-4}$, albeit at higher cost. The leftmost black lines represent fundamental performance limits with perfect particles and discrimination distribution $\left({\rm{\sigma }}=0,{\rm{\lambda }}=0\right)$ – this corresponds to the stochastic sensing limit that no assay can surpass. The overlap between monomer and dimer intensity values $\left({\rm{\sigma }}\right)$ was not as impactful as the monodispersity of the initial particle distribution (low ${\rm{\lambda }}$). For example, even if monomer and dimer intensities have 25% overlap in their distributions, subfemtomolar concentrations can be reliably detected with 100 images worth of particles. We note that atto- or zeptomolar detection limits can be achieved by reducing the total number of particles (Fig. 5d). This increases the fraction of particles that capture analyte but comes at a cost of longer incubation times.

## Discussion

Here we examined two different ML strategies for improving image-based assay performance: a hybrid approach combining pixel segmentation with traditional image processing and an end-to-end deep learning model that directly mapped images to analyte concentrations. While both methods improved over conventional assays, the latter significantly outperformed the hybrid approach in terms of sensitivity and robustness. The hybrid approach effectively removed background noise and extracted useful nanoparticle properties, but it was inherently limited by predefined feature selection. The end-to-end classification approach, by contrast, leveraged all available image features and dynamically learned which properties were most indicative of analyte concentration. This ability to self-correct and learn from diverse sources of noise led to notable improvements in dynamic range, sensitivity, reproducibility, and general tolerance to error and unskilled handling. ML training was accomplished with moderately sized data sets, that can be easily collected in a manner of hours with simple laboratory equipment, and a single GPU. It is entirely practical for specific ML algorithms to be trained for each particle batch on a wide range of imaging platforms.

This study introduces an ML-driven approach to digital molecular assays that significantly enhances overall performance while reducing hardware complexity. By leveraging image-based analysis and deep learning, we demonstrate a system that outperforms both ELISA and LFA in key metrics such as sensitivity, dynamic range, and robustness to error. This advancement represents a critical step toward more accessible, reliable, and scalable diagnostic solutions, particularly for POC and self-administered testing applications. These advancements were achieved through a limited application of modern machine learning tools. We can expect that more sophisticated approaches will lead to further progress. As the field of digital molecular diagnostics continues to evolve, the integration of computational intelligence with molecular assays holds immense potential to transform healthcare by making high-quality diagnostics more widely available.

## Methods

### Gold nanoparticle conjugation and characterization

Carboxylated gold nanoparticles (AuNPs) with a nominal diameter of 80 nm (NanoComposix, SKU: AUXR80-5M; optical density 20, corresponding to approximately 1.3 × 10¹¹ particles mL⁻¹) were used as the signal reporters for all assays. Nanoparticles were functionalized with antibodies or recombinant proteins using carbodiimide-mediated amide coupling, followed by surface passivation to ensure colloidal stability in serum and whole blood.

For C-reactive protein (CRP) assays, two monoclonal anti-CRP antibodies recognizing distinct epitopes (anti-C2, Abcam ab17452; anti-C6, Abcam ab244707) were conjugated to AuNPs to generate a two-capture sandwich format. For anti-SARS-CoV-2 IgG serology assays, recombinant receptor-binding domain (RBD) protein from the SARS-CoV-2 spike protein (Abcam AB273065-1003) was conjugated to AuNPs to produce single-capture particles specific for anti-SARS-CoV-2 immunoglobulins.

Immediately prior to conjugation, fresh solutions of 1-ethyl-3-(3-dimethylaminopropyl) carbodiimide (EDC) and sulfo-N-hydroxysuccinimide (sulfo-NHS) were prepared at 10 mg mL⁻¹ in deionized water. For a standard 1 mL conjugation reaction, 7 µL of EDC solution was added to 1 mL of carboxylated AuNPs suspended in deionized water, followed by 14 µL of sulfo-NHS solution. The mixture was gently mixed and incubated for 30 min at room temperature with rotation to activate surface carboxyl groups.

Activated particles were collected by centrifugation at 5000 × *g* for 5 min. The supernatant was carefully removed, and the pellet was resuspended in 1 mL of reaction buffer consisting of 5 mM KH₂PO₄ supplemented with 0.5% (v/v) 20 kDa PEG at pH 7.4. Particles were resuspended by brief vortexing and mild sonication (< 30 s) to disrupt loose aggregates without damaging surface chemistry.

Protein conjugation was performed by adding capture protein to the activated particles at a final amount of 20 µg per mL of nanoparticle solution (e.g., 20 µL of a 1 mg mL⁻¹ protein stock). The reaction was incubated for 2 h at room temperature to allow covalent amide bond formation. Unreacted NHS esters were subsequently quenched by addition of 5 µL saturated glycine solution (prepared in deionized water), followed by incubation for an additional 10 min.

Following conjugation and quenching, particles were washed by centrifugation (5000 × *g*, 5 min) and resuspended in fresh reaction buffer. This wash step was repeated twice to remove unconjugated protein and reaction byproducts. After the final wash, particles were resuspended in 0.1× PBS supplemented with 0.5% (v/v) Tween-20.

To further stabilize particles and refill unoccupied surface sites, a secondary PEGylation step was performed. Thiol-terminated PEG with terminal carboxyl groups (SH-PEG-COOH, MW 6,000) was dissolved at 40 µg mL⁻¹ in 0.1× PBS. Equal volumes of PEG solution and protein-conjugated AuNPs were mixed and incubated for 6 h at 25 °C with gentle shaking, or overnight at 4 °C. Particles were then washed three times by centrifugation and resuspension in 0.1× PBS containing 0.5% Tween-20 and stored at 4 °C until use.

Successful conjugation and functional activity were confirmed by analyte-dependent nanoparticle aggregation observed under darkfield microscopy. Functionalized particles remained colloidally stable in buffer and biological matrices in the absence of target analyte and exhibited robust aggregation only upon specific target binding. No additional size-exclusion chromatography or affinity purification was performed.

### Colorimetric bulk assays

CRP detection using bulk absorbance was performed by incubating anti-CRP AuNPs with recombinant CRP (Abcam AB283925-1003) at various concentrations (0.78–166 mg L⁻¹). After incubation for 1 h at room temperature, UV-Visible absorbance spectra were collected using a spectrometer. Absorbance at 555 nm was quantified and normalized to control samples lacking CRP. Peak shifts and aggregation signatures were seen in the spectra across 500–700 nm.

### Imaging and sample preparation

Imaging experiments were performed using an Olympus BX43F upright microscope equipped with a darkfield condenser and an SC180 CMOS camera. Images were acquired using a 20× objective (numerical aperture specified by manufacturer) under darkfield illumination, which enables direct visualization of individual gold nanoparticles and nanoparticle clusters against a dark background. Illumination was provided by the microscope’s standard transmitted light source (at max illumination intensity) and held constant across experiments. Camera exposure time, gain, and bit depth were fixed for each experiment to ensure consistent image statistics; images were recorded as uncompressed files without post-acquisition normalization unless otherwise noted.

For imaging assays, samples were prepared by mixing 5 µL of biological sample (serum, plasma, or whole blood) with 2 µL of functionalized gold nanoparticle solution and 18 µL of 0.1× PBS, yielding a final assay volume of 25 µL. Samples were gently mixed by pipetting and incubated for 30 min at room temperature (22–24 °C) without agitation to allow nanoparticle–analyte binding and aggregation to reach quasi-equilibrium. This incubation time was selected to exceed the characteristic on-rates of the capture reagents used while remaining compatible with rapid point-of-care workflows.

Following incubation, 6 µL of the assay mixture was transferred to a standard 75 × 25 mm glass microscope slide and covered with a 18 mm square coverslip (No. 1.5 thickness). Samples were imaged immediately after mounting to minimize sedimentation and evaporation effects. Imaging fields of view were selected to avoid visible edges of the coverslip or gross defects in the glass substrate. For each sample, multiple non-overlapping fields of view were acquired to capture a representative distribution of nanoparticles and aggregates.

For experiments involving whole blood, commercially sourced human blood (Fisher Sci NC1570874) was used as received with the supplier-specified anticoagulant. Whole blood samples were spiked with recombinant analyte (CRP: Abcam AB283925-1003) or antibody standards (IgG: Thermofisher MA535939) as described in the corresponding assay sections and incubated directly with functionalized nanoparticles without plasma or serum separation. Whole-blood imaging produced additional optical artifacts, including hemoglobin-induced color shifts, red halo effects, and increased background scattering from blood cells. These effects were intentionally retained in the dataset to evaluate algorithm robustness under realistic, high-error conditions. The whole-blood CRP data and whole-blood IgG data were collected on different microscopes and with different imaging substrates (the former in square capillary tubes and the later on microscope slides) which is why RBCs are more easily resolved in the IgG experiments (Fig. 2b).

In selected experiments, samples were also imaged in open-air microwell formats rather than under coverslips. These microwells introduced additional sources of optical and geometric variability, including meniscus-induced distortion, variable sample thickness, dust contamination, and bubble formation. These conditions were used deliberately to stress-test both conventional image analysis pipelines and machine-learning–based approaches, and do not represent an optimized assay configuration.

All image analysis was performed on raw images unless explicitly stated otherwise. Images shown in figures were contrast-enhanced for visualization purposes only and were not used for quantitative analysis. The number of images acquired per sample and the distribution of fields of view were chosen to balance statistical sampling of particle populations against practical imaging throughput.

### Hybrid pixel-wise segmentation and traditional image processing

The pixel segmentation model we used was a Unet trained on a collection of images, each labelled by one of 7 random forest classifier (RFC) models created using ilastik. Training images for the RFCs included nanoparticle clusters and background elements such as blood cells, bubbles, and other artifacts. The RFC models used by ilastik perform well on homogeneous datasets but fail to generalize across broader sets of images. As such, sets of 5–10 images were grouped to maintain RFC performance and 7 separate RFC models were trained spanning the diversity of images in the dataset. We note that training RFCs with several classes (background, particles, blood cells, bright debris, scratch, etc.) outperformed a simple background/foreground approach. In images involving human blood (Figs. 3b), 1 µg of recombinant anti-SARS-CoV-2 antibody (Thermofisher MA535939) was spiked into 1 mL of whole blood (Fisher Sci NC1570874).

A Unet was subsequently trained on the pixel classified images using standard data augmentation techniques. Once the Unet was trained, images were segmented by their predicted probability for pertaining to the particle class. Post-processing of the probability maps included morphological filtering, size exclusion, watershed filtering, and shape-based filtering to separate distinct objects. Cluster metrics such as mean area, brightness, and shape descriptors were extracted using the scikit-image library for Python.

### End-to-End deep learning model training

An end-to-end image regression model was developed using a ResNet-18 backbone via the PyTorch library: torchvision.models.resnet18. A total of 10,000 darkfield images from 182 samples were used to train the model, with sample concentrations titrated across 7 levels (0 to 16384 ng/µL). Data was split into training (164 samples) and testing (18 samples) sets by sample, ensuring no data leakage due to repeated, albeit non-overlapping, imaging of the same specimen. The model was trained on unnormalized images. The final regression layer output a single scalar corresponding to the natural logarithm of concentration, the zero concentration was labelled as *−*0.6931 while the second lowest concentration (16 ug/mL) was labelled as 2.773. Model performance was validated on a separate test set using the mean prediction from 20 images per sample.

### Clinical serum samples

*Ethics and Biospecimens*. Human serum specimens (*n* = 56) were obtained from Precision for Medicine (Norton, MA) as de-identified biobanked specimens. This cohort consisted of 30 clinical COVID-19 positive serum samples and 26 pre-COVID-19 negative control samples. Precision for Medicine certified that all biospecimens were collected from living donors via venous blood draw (typically from the antecubital vein) under a clinical study approved by an Institutional Review Board (IRB) and/or Independent Ethics Committee (IEC) in accordance with the Declaration of Helsinki and applicable local regulations (45 CFR 46; 21 CFR 56). The protocol name was P4M060 under Advarra IRB. Informed consent was obtained (21 CFR 50), or specimens met FDA criteria (2006) for de-identified remnant specimens. The positive samples were collected between October 2020 and February 2021 from donors with PCR-confirmed SARS-CoV-2 infection (7–21 day positive PCR testing). Control samples were collected between October 2018 and January 2019. Blood samples were stabilized via centrifugal separation into 1 mL serum aliquots and maintained in long-term frozen storage at ≤−20 °C prior to shipment. Positive specimens were selected for this study based on confirmed PCR diagnostic results and a minimum required aliquot volume of 1 mL. The authors did not have access to identifiable private information.

BRISQ Tier 1 Guidelines Table:

**Table:** 

Item #	Data Element	Reported Information / Status
I.a.	Biospecimen type	Human serum specimens (*n* = 56 total).
I.a.1.	Anatomical/Collection site	Venous blood draw (typically from the antecubital vein).
I.a.2.	Biospecimen disease status	30 COVID-19 positive samples; 26 pre-2019 negative control samples.
I.b.	Clinical characteristics	Positive Cohort: Donors with confirmed PCR results (predominantly 7–21 days post-test). Negative Cohort: Mixed clinical profiles including Type II Diabetes, Hypertension, and Asthma; includes “never,” “former,” and “current” smokers.
I.b.1.	Vital state	Specimens were collected from living donors.
I.b.2.1.	Diagnosis (Clinical)	Positive Cohort: PCR-confirmed COVID-19. Negative Cohort: Inferred negative status based on collection date (October 2018 – January 2019) prior to known SARS-CoV-2 circulation.
I.b.2.2.	Diagnosis (Pathology)	N/A (Liquid biopsy/serum study).
II.a.	Collection mechanism	Venous blood draw.
III.a.	Stabilization mechanism	Centrifugal separation.
III.b.	Long-term preservation	Frozen storage.
III.b.1.	Constitution of preservative	None (1 mL serum aliquots).
IV.a.1.	Storage temperature	Maintained frozen at ≤ -20 °C.
IV.a.2.	Storage duration	Approximately 1–3 years (Collected 2018–2021; analyzed in 2022).
IV.b.1.	Shipping temperature	Frozen; shipped via FedEx Priority Overnight on 2/9/2022.
V.a.	Composition & Selection	Selected based on PCR result or collection date (pre-2019 for controls), and 1 mL aliquot availability.

For the classification experiment (using segmentation + traditional feature analysis), two images of each specimen were analyzed in triplicate wells (6 total images) per sample and imaged after direct addition of nanoparticle mixture as described in above in sample preparation. Performance metrics such as positive predictive value and false positive rate were calculated from the model outputs.

### ELISA and lateral flow assays

The ELISA comparison was done with a Human SARS-CoV-2 Spike (trimer) IgG ELISA Kit from Thermofisher (BMS2325). Assays were performed following manufacturer instructions. Absorbance was read at 450 nm using a microplate reader. The limit of detection and dynamic range were calculated from the standard curve. A lateral flow assay was assembled in-house using a Universal LFA kit (Abcam AB270537-1001) and the above described RBD-functionalized AuNPs as reporters. Test strips were imaged using a laboratory scanner (Azure Biosystems 600), and band intensity was quantified using python.

### Monte Carlo hypothesis testing

Within the linear range of camera detection, particle intensities tend to convolve under aggregation (dimer intensities are the auto-convolution of the monomer distribution). Baseline particle-intensity distributions contain a mixture of monomer, dimer, trimer, and other higher order species. To model the baseline intensity distribution simply, we chose to approximate the distribution as a mixture of normal distributions (as normal distributions are closed under convolution). For naïve crosslinking, a particle will participate in j crosslink events according to a binomial distribution, and since $N$ is large ( > 1 million) the probability tends to be small ( < 0.1), so we chose Poisson weightings for the monomer, dimer, etc. component distributions. Thus, we created a baseline distribution with the form: $B\left({{\rm{\lambda}}},{\mu},{\sigma}\right)={\sum}_{i=1}^{i=20}{{\rm{Poisson}}}\left(i-1;{\rm{\lambda }}\right){\rm{Normal}}\left(i{\mu},i{\sigma}\right)$. Here, ${\rm{\lambda }}$ captures the polydispersity of the baseline particles. Lower ${\rm{\lambda }}$ results in greater monomer. Next, we introduced a cross-linking rate $k$, ranging from 0.1 down to 1e-8. To “crosslink” the baseline distribution we first convolved the baseline distribution with itself 4 times and then created a new mixture distribution $M\left(k,{\rm{\lambda }},{\mu},{\sigma}\right)={\sum }_{j=1}^{4}{{\rm{Poisson}}}\left(j-1;k\right){B}_{j}\left({{\rm{\lambda }}},{\mu},{\sigma}\right)$, where ${{B}_{j}}\left({{\lambda }},{{\mu }},{{\sigma }}\right)$ is the j-th convolution of the baseline with itself. Only four self-convolutions were considered since the coefficients of the normal-components corresponding to higher-order aggregates quickly dropped off below 1e-12.

To perform the hypothesis testing, 300 replicates of sample size n (varying from 1e1 to 1e8) were drawn from the baseline $B\left({\rm{\lambda }},{\rm{\mu }},{\rm{\sigma }}\right)$ and crosslinked distribution $M\left(k,{\rm{\lambda }},{\rm{\mu }},{\rm{\sigma }}\right)$. The log-likelihood ratio, $\Lambda$, was computed on both B and M samples, producing null and alternative distributions of the log-likelihood ratio statistic. A Type I error ${\rm{\alpha }}$ level of 0.05 was used on the baseline B samples; meaning, a threshold on $\Lambda$ was chosen such that only 5 percent of samples from B were larger than said threshold. Next, the Type II error rate ${\rm{\beta }}$ of the threshold was computed to be the fraction of samples from M producing $\Lambda$ below the threshold. If the power $\left(1-{\rm{\beta }}\right)$ was below 0.95, then a larger sample size (*n*) was attempted until sufficient power was reached.

The crosslinking rate, $k$, is approximately the fraction of higher order species. Since our experiments used approximately 100 million particles per sample, the crosslinking rate was converted to concentration by assuming perfect crosslinking and k x 100 million crosslinkers in 25 µL. For imperfect crosslinking, for example a 1 pM binder, then using a simplified equilibrium analysis with excess capture ligand, would mean that each crosslinker has a $> 99 \%$ probability of being bound, thus mildly shifting the scaling curves to the right.

### Statistical analysis

All data analysis was performed using Python (NumPy, SciPy, scikit-image, and PyTorch). Unless otherwise stated, data are reported as mean ± standard error of the mean (SEM). Each reported concentration represents measurements from independent biological samples, with multiple non-overlapping image fields acquired per sample.

For image-based assays, analyte concentration estimates were derived from either aggregate image metrics (hybrid analysis) or the mean prediction across multiple images per sample (end-to-end regression). Sample-level predictions were computed by averaging model outputs from 20 images to reduce field-of-view variability.

Limits of detection (LOD) were estimated from Hill functions using “blank” + 2σ of measurement. The limit of saturation (LOS) was estimated from Hill functions using the “fitted saturation level” – 2σ of measurement. Dynamic range was defined as the log10(LOS/LOD).

For clinical classification experiments, positive predictive value (PPV) and false positive rate were computed using standard definitions, but did not include a priori prevalence weightings. Monte Carlo hypothesis testing was used to estimate sensitivity scaling laws, with significance level α = 0.05 and statistical power (1 − β) ≥ 0.95.

No statistical methods were used to predetermine sample size. No data points were excluded unless explicitly stated.

## Supplementary information

Supplementary Information

## Data Availability

The datasets, code, and custom materials generated and/or analyzed in this study are available to qualified researchers or organizations upon reasonable request, subject to the execution of an appropriate legal agreement (e.g., non-disclosure agreement or material transfer agreement) between the requesting party and ilytica, LLC. Requests for access should be directed to darren@ilytica.com mailto:darren@ilytica.com or jtgroves@lbl.gov mailto:jtgroves@lbl.gov.
