# Supplementary material for: Homogeneous image-based digital immunoassays with high error tolerance
Source: Npj Imaging. 2026 May 4;4:30. doi: 10.1038/s44303-026-00164-9 (PMC13139397; doi:10.1038/s44303-026-00164-9)
Supplement: Supplementary file 1 — Supplementary Information [file 44303_2026_164_MOESM1_ESM.pdf]

Supplemental Information

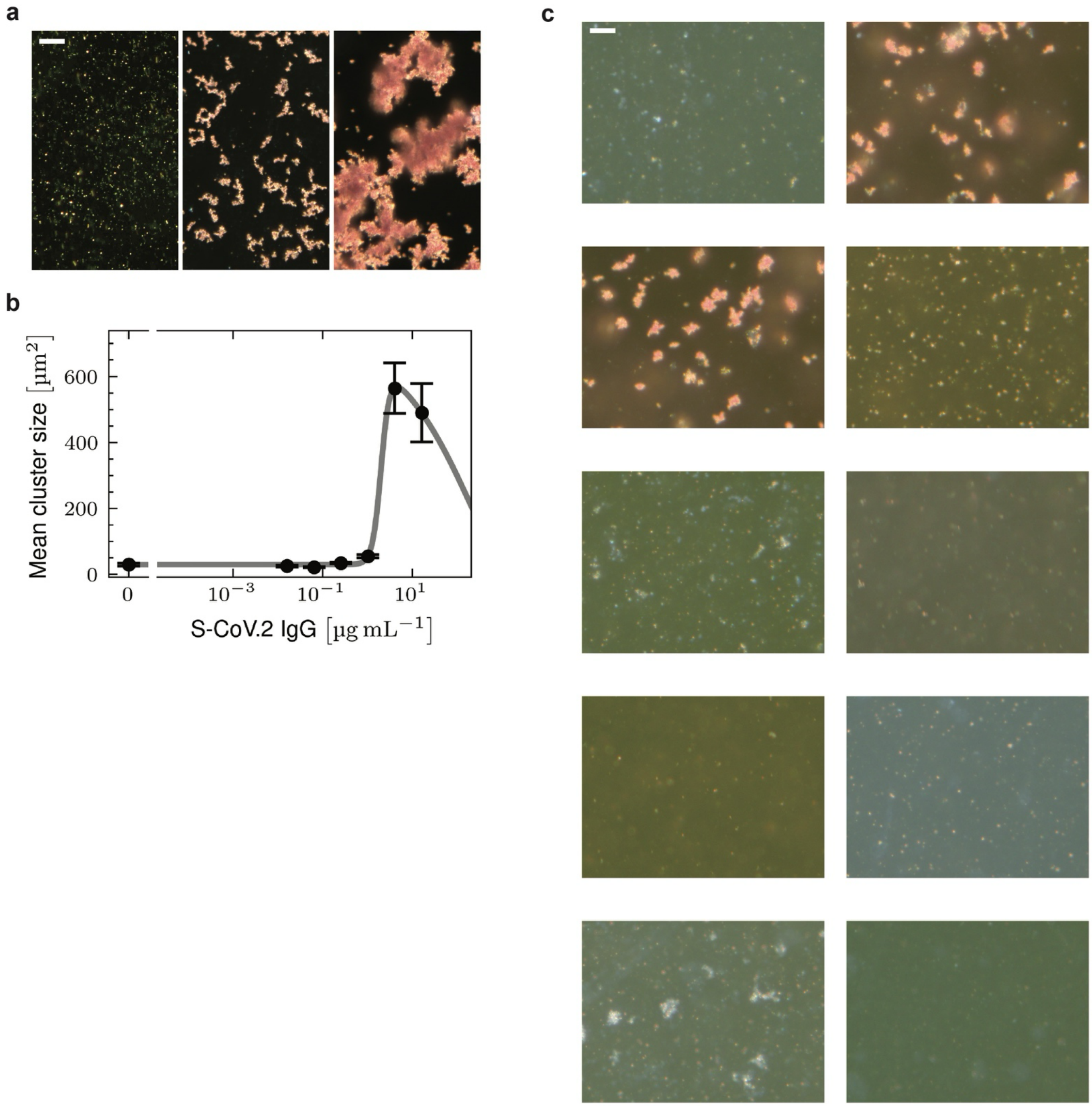

Supplemental Figure 1 **Single-species aggregation assay and example images with significant error. (a)** Example images of particle aggregation in the presence of increasing antibody (Ab against S-CoV.2 RBD). 20x darkfield was used. **(b)** Quantification of cluster size as a function of S-CoV.2 IgG concentration. **(c)** Example images taken using 20x darkfield in open air microwells with notable optical artifacts. Scale bars are 15 μm.
